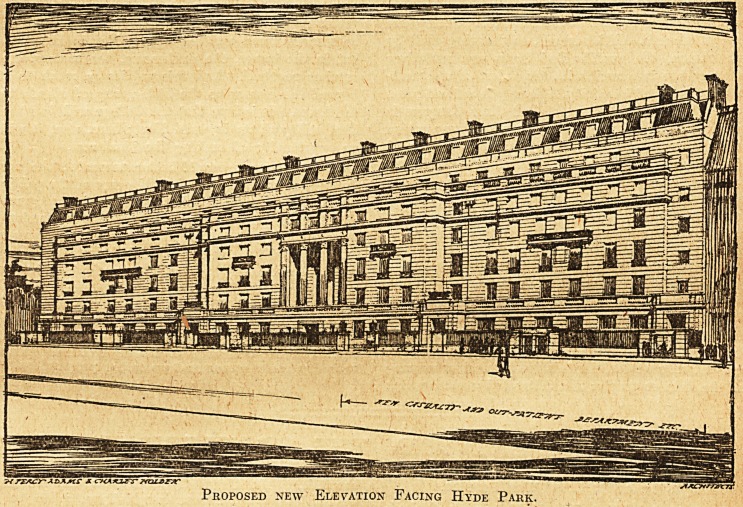# New Building Decided on

**Published:** 1919-04-19

**Authors:** 


### ST. GEORGE'S HOSPITAL.

### New Building Decided on.

The Governors of St. George's Hospital have definitely decided to retain the Hospital at Hyde Park Corner but it has been recognised, for some considerable time, that the accommodation is, in many respects, inadequate for the work that has to be carried on. This is especially noticeable in the cramped out-patient and casualty departments, and great inconvenience in administration is also caused by the housing of the nurses at a distance from the hospital. The necessary alterations have been postponed from year to year but it has now been decided to erect a new building upon the site of the present officers' residence and of the four houses in Knightsbridge adjoining and belonging to the hospital, to provide new casualty and out-patient departments and officers' and nurses' quarters, which building is to be so arranged that it may form part of a future new hospital. Plans have been prepared and some idea of the improvement may be derived from the illustration, which shows the intended elevation facing Hyde Park; the portion now proposed to be erected is that part to the west of the central feature.

The building will contain on the ground floor the casualty department and in the basement the out-patient department consisting of a waiting-hall for 250 patients. Arranged round this will be the medical, surgical, obstetrical, ophthalmological, aural and dental consulting rooms, operating-theatre, and also the dispensing and almoner's departments. On the first and second floors will be the medical officers' quarters and on the four upper floors accommodation for over 100 nurses. The building is to be faced with Portland stone.

The Committee hopes to be able to proceed with the working drawings as soon as Peace is proclaimed but does not intend to start actual building until prices have moderated and conditions justify this step being taken.

Proposed new Elevation Facing Hyde Park.

The needs of the hospital are greater than ever they were. Expenditure increased in 1918 to £59,592, whilst the ordinary income was £40,250, leaving a deficit of £19,342, which had to be met by utilising the legacies received in cash during the year and by realising a certain amount of capital, thus reducing the certain income. Also, during the war, a number of subscribers have died and it is imperative that they should be replaced by others. Therefore those who have the interests of the hospital at heart must not slacken in their efforts.

The Committee have made a tangible increase in the salaries of the nursing staff, which involves an addition of £300 per annum to the expenditure.

The work and expenses of the hospital have naturally increased with the formation of new departments, *i.e.*, tuberculosis, venereal diseases, etc, 146 new cases having been seen in the tuberculosis dispensary and 397 in the venereal diseases department. Another branch of the work which is most expensive is that connected with electro-therapeutic treatment, and about 1,800 cases are seen annually in this department.

The number of military patients received into the hospital during 1918 was 414, making a total of 2,052 since the commencement of the war, and it is a source of much gratification to the Committee that there have been only twenty-eight deaths amongst them—twenty-one as the result of war service and seven from natural causes—particularly as the cases selected to be sent have been of a serious nature.

The average number of beds occupied during the past year by civilian cases has been 235, and by military cases 65, the total number of beds in the hospital being 341. In all 4,254 in-patients and 29,073 out-patients have been treated.

## Figures and Tables

**Figure f1:**